# Analysis of Musculoskeletal Injuries Among Collegiate Varsity Electronic Sports Athletes

**DOI:** 10.7759/cureus.31487

**Published:** 2022-11-14

**Authors:** Ari J Clements, Ryan W Paul, Adam J Lencer, Daniel A Seigerman, Brandon J Erickson, Meghan E Bishop

**Affiliations:** 1 Orthopaedic Surgery, Thomas Jefferson University, Philadelphia, USA; 2 Sports Medicine, Rothman Orthopaedic Institute, Philadelphia, USA; 3 Sports Medicine, Philadelphia College of Osteopathic Medicine, Philadelphia, USA; 4 Orthopaedic Surgery, Rothman Orthopaedic Institute, New York, USA; 5 Sports Medicine, Rothman Orthopaedic Institute, New York, USA

**Keywords:** electronic sports, overuse, collegiate, musculoskeletal, injury, esports

## Abstract

Background

Collegiate electronic sports (esports) in the United States has grown from seven varsity programs in 2016 to over 200 today. Despite its growing success, little has been studied on the injuries of these athletes. In this study, we aimed to investigate the prevalence of injuries sustained by collegiate esports players and explore the injuries’ impacts on their careers. The authors hypothesized that athletes who spend more time practicing and playing competitively in esports will report an increased history of musculoskeletal injury.

Methodology

This was a cross-sectional study, level IV evidence. A list of collegiate esports athletes was collected from publicly available sources. Athletes with available contact information were sent a self-reporting questionnaire. The questionnaire examined variables including length of time playing esports, hours/day playing esports, esports-related injury history, surgeries needed, and missed competition time.

Results

Overall, 153 collegiate esports athletes (88% male, aged 18-42 years) were included, with 41 (26.8%) having experienced at least one injury from esports. Of the 41 injured athletes, three (7.3%) required surgery, 17 (41.5%) had multiple injuries, and seven (17.1%) missed competition time for an average of 3.0 ± 2.3 weeks. Athletes who have been on their respective college team longer (2.0 ± 1.0 vs. 1.7 ± 0.9 years, p = 0.03) and who spent more hours per day practicing had a higher injury incidence (p = 0.01). There was no difference in the current age, sex, age at which athletes began competing in esports, and scholarship status between groups (all p > 0.05). When analyzing the 41 athletes who experienced an injury, the most common injury was to the wrist with 25 total injuries. There were 11 neck, 10 back, nine finger, eight hand, six elbow, and four thumb injuries.

Conclusions

Collegiate esports players who trained for more hours per day (31.7% of injured players vs. 10.7% of uninjured players practiced more than five hours/day, p = 0.01) and played competitive collegiate esports for more years (2.0 ± 1.0 vs. 1.7 ± 0.9 years, p = 0.03) were more likely to have experienced an esports-related injury. Fortunately, only a small portion of athletes who experienced an injury was forced to miss competition time or require surgery. With this being the largest investigation into collegiate esports-related injuries, future medical research regarding the incidence, management, and prevention of its injuries can help collegiate and professional programs place a greater emphasis on the health of their athletes.

## Introduction

Electronic sports (esports) have grown tremendously in the past decade from an estimated market value of $493 million in 2017 to an expected growth of $2.3 billion dollars by the end of 2022 [1]. Colleges and universities are embracing this growing culture with over 200 varsity esports programs in the United States as of May 2022, up from seven varsity programs two years prior [2].

Esports players report practicing for an average of 3-10 hours per day, and 40% of players report a lack of physical exercise, thus putting themselves at higher risk for repetitive stress injuries [3]. Prior studies have noted that injuries to professional esports players have forced them to miss significant time and, in some instances, into early retirement [4]. The most common injuries to esports athletes include injuries to the hand, wrist, back, and neck [5].

Several studies have investigated excessive video game playing among young athletes and found that excessive video game playing was associated with musculoskeletal pain including low back pain [6,7]. However, television viewing alone was not associated with the same symptoms [7]. Sekiguchi et al. investigated the impact of video game playing among 200 9-12-year-old baseball players, finding that playing video games for three or more hours per day was significantly associated with elbow or shoulder pain [8]. Because the increase in sedentary lifestyles and video game playing in children will only increase total participation in esports, there should be a continued emphasis on the health and injury prevalence of its athletes.

While esports injuries have not been frequently reported, the impact of these injuries cannot be understated as they have made professional gamers miss championship matches, forced early competitive retirement, and led to lingering musculoskeletal injuries. Therefore, the purpose of this study was to investigate the prevalence of injuries sustained by collegiate esports athletes. Our hypothesis is that athletes who spend more time practicing and playing competitively in esports will report an increased history of musculoskeletal injury.

## Materials and methods

Inclusion/Exclusion criteria

This cross-sectional study was approved by the Thomas Jefferson University Institutional Review Board (#19D.743) prior to survey administration. Of the 202 colleges and universities with esports programs, only schools that have publicly available contact information were contacted for participation [9]. Thus, all current esports athletes who completed the survey were included. Athletes were excluded if they were not current collegiate esports athletes or if the survey was not fully completed.

Data collection

The survey evaluated the following variables: age, sex, duration of sport participation, training workload, injuries to various body regions, surgery due to injury, and competition time missed due to injury. Anonymous survey responses were compiled using RedCap (Vanderbilt University, Nashville, Tennessee) and Microsoft Forms (Redmond, Washington). Athletes with available email addresses were contacted by email and program directors with available phone numbers or email addresses were also called to request that they encourage participation from the athletes.

Statistical analysis

Demographic and workload data were compared between injured and uninjured athletes. Parametric continuous data are presented as mean (standard deviation), and p-values were calculated by performing t-tests. Nonparametric continuous data are also presented as mean (standard deviation) for easier interpretation and calculated by performing Mann-Whitney tests. Categorical data are presented as the sample size (%). Chi-square or Fisher’s exact tests were used to calculate p-values for categorical data. P-values less than 0.05 were deemed significant. A spearman correlation coefficient was used to evaluate the relationship between days per week practicing and injuries. All statistical analyses were done using R Studio (version 3.6.3, Vienna, Austria).

## Results

Overall, 153 collegiate esports athletes responded to the survey, with the cohort consisting of 135 (88.2%) males and an average age of 21.0 ± 3.6 years (range = 18-42 years). Overall, 41 (26.8%) of the included athletes experienced at least one injury from esports, and 17 (11.1%) had multiple injuries. Of the 41 injured athletes, three (7.3%) required surgery, and seven (17.1%) missed competition time for an average of 3.0 ± 2.3 weeks (range = 1-8 weeks).

Athletes who had been on their respective college teams longer had a higher injury incidence such that injured athletes spent 2.0 ± 1.0 years on the team compared to 1.7 ± 0.9 years for uninjured athletes (p = 0.03). Additionally, athletes who spent more hours per day practicing had a higher injury incidence. Specifically, 13 of the 41 injured athletes (31.7%) practiced for more than five hours per day as opposed to only 12 of the 112 uninjured athletes (10.7%) who reported practicing more than five hours per day (p = 0.01) (Table 1). There was no difference in the current age, sex, age at which athletes began competing in esports, and scholarship status between groups (all p > 0.05).

**Table 1:** Comparison of demographics between injured and uninjured esports athletes. Categorical data are presented as n (%), and continuous data are presented as mean ± standard deviation. Statistically significant differences are in bold.

Demographic	Uninjured (n = 112)	Injured (n = 41)	P-value
Current age (years)	20.7 ± 2.7	21.8 ± 5.3	0.32
Sex (% male)			0.08
Male	92.0%	80.5%	
Female	8.0%	19.5%	
Age started competing in esports (prior to college)	16.3 ± 3.5	16.4 ± 4.2	0.87
Years on the collegiate team	1.7 ± 0.9	2.0 ± 1.0	0.03
Scholarship (% on scholarship)	33.0%	39.0%	0.62
Hours per day playing esports			0.01
0–1 hour	4.5%	0.0%	
1–2 hours	19.6%	14.6%	
2–3 hours	29.5%	39.0%	
3–4 hours	22.3%	9.8%	
4–5 hours	13.4%	4.9%	
5+ hours	10.7%	31.7%	
Days per week playing esports	4.7 ± 1.5	4.5 ± 1.5	0.49

**Figure 1:**
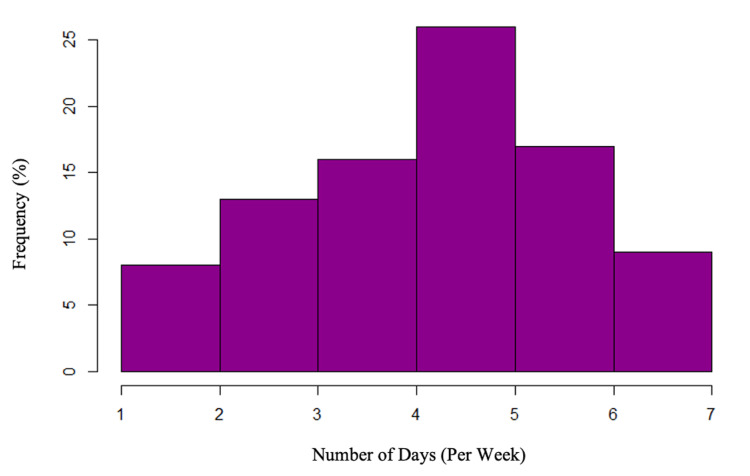
Distribution of days per week practicing esports.

Likewise, there was no significant difference in days per week playing esports between uninjured and injured athletes (4.7 ± 1.5 vs. 4.5 ± 1.5 weeks, p = 0.49) (Figure 1). As demonstrated by the Spearman correlation coefficient for days per week practicing and injuries sustained of -0.07, there was no significant correlation between days per week workload and injury.

The most commonly reported injury was to the wrist. Of the 75 injuries to the 41 athletes, there were 25 wrist injuries (33.3%), 11 neck injuries (14.7%), 10 back injuries (13.3%), nine finger injuries (12.0%), eight hand injuries (10.7%), six elbow injuries (8.0%), four thumb injuries (5.3%), and two shoulder injuries (2.7%) (Table 2).

**Table 2:** Location of the 75 observed injuries sustained by esports athletes.

Injury type	Quantity of injuries	Percentage of 75 total injuries
Wrist	25	33.3%
Neck	11	14.7%
Back	10	13.3%
Finger	9	12.0%
Hand	8	10.7%
Elbow	6	8.0%
Thumb	4	5.3%
Shoulder	2	2.7%

Of the seven athletes who missed time due to injury, three (42.9%) had multiple injuries and one (14.3%) was on scholarship. Cumulatively, there were 12 injuries that resulted in time out of competition: three wrist injuries, three finger injuries, two elbow injuries, two thumb injuries, one hand injury, and one back injury (Table 3).

**Table 3:** Descriptions of the participants who experienced an injury that led to missed competition time.

Participant	History	Injury	Time missed
20-year-old female	Started playing at 18, plays 2–3 hours per day for 2 days per week	Right wrist	2 weeks
24-year-old male	Started playing at 18, plays 5+ hours per day	Finger, wrist, elbow	2 weeks
19-year-old male	Started playing at 13, on scholarship, plays 5+ hours per day	Back	2 weeks
22-year-old male	Started playing at 17, plays 5+ hours per day	Finger, hand	8 weeks
24-year-old male	Started playing at 18, plays 5+ hours per day	Finger, wrist, elbow	2 weeks
42-year-old male	Started playing at 20, plays 2-3 hours per day	Thumb	3 weeks
40-year-old male	Started playing at 18, plays 2-3 hours per day	Thumb	3 weeks

## Discussion

This study was conducted to serve as a foundation for injury identification, prevention, and management in the rapidly growing esports industry. By understanding the common practices of competitive esports gaming and the patterns of injuries, medical professionals can better guide esports players as participation continues to increase. The authors’ hypothesis was correct in that both an increase in the hours per day that a player practices and a longer duration on their team increased the risk of injury. Interestingly, while hours per day was a significant risk factor for injury, the number of days per week was not a significant risk factor for injury, possibly indicating that injuries are sustained through continuous and excessive playing rather than intermittent playing over a longer period.

With an increase in esports popularity, revenue, and international participation, research into esports-related injuries and their prevention will be key for both collegiate and professional programs. In 2017, Goldman Sachs estimated that with an almost doubled increase in audience, player sponsorships, and advertising/media rights, the esports industry can grow to upwards of $3 billion [10]. Newzoo, a games market analytics company, projected that in 2022 the esports market audience will reach 532 million people globally, an 8.7% increase year-on-year [11]. In the past several years, esports has also gained recognition as an organized sport by several large organizations; for example, the International Olympic Committee recently created the Olympic Virtual Series, which is a series of virtual physical and non-physical events leading up to the 2021 Tokyo Olympic games that may be part of Olympic games in the future [12].

Lindberg et al. analyzed the impact of esports-related pain, reporting a negative association between musculoskeletal pain and subsequent esports playing time during the weeks with pain [13]. This finding is important in proving that players will decrease their playing and potentially competition time if they sustain an injury. Our study found that with the same amounts of days per week, 31.7% of the injured athletes practiced for more than five hours per day but only 10.7% of uninjured athletes practiced for more than five hours per day; hence, an increased playing load is associated with three times higher injury risk. These two findings together demonstrate that players who practice for more than five hours per day are putting themselves at risk for injury and decreased playing/competition time in the future. Further research is needed for a recommendation on the optimal number of hours per day playing esports without having an increased risk for injury.

While our study primarily assesses the impact the injuries may have on the players’ careers, we also sought to examine the patterns of esports-related injuries. To date, there are only two studies assessing the prevalence of esports injuries. DiFrancisco-Donoghue et al. surveyed 65 collegiate esports athletes from eight universities [3]. Their results revealed that the athletes practice 5.5-10 hours per day with 52% reporting eye fatigue, 41% reporting back or neck pain, 36% reporting wrist pain, 30% reporting hand pain, and only 2% seeking medical attention [3]. Lindberg et al. found that 42.6% of the 188 esports athletes who responded to their survey reported musculoskeletal pain, with the back (31.3%), neck (11.3%), shoulder (11.3%), wrist (6.3%), and hand (5.0%) being the most common sites of injury [13]. Compared to these studies, our study’s prevalence of 26.8% may even present an underrepresentation of the actual injury prevalence. This difference could be explained by the wording of surveys. While both of the other studies assessed for musculoskeletal “pain,” our study specifically asked for “injuries,” which is seemingly more restrictive.

Nevertheless, all three studies indicated that wrist, back, and neck pain were three of the most problematic areas for esports players. Similar to occupations that require long periods of using computers or consoles, the players’ static postures, repetitive and forceful motions, and prolonged sitting are all risk factors for poor musculoskeletal outcomes [14]. As McGee et al. note, both populations suffer neuropathic and tendinopathic conditions, specifically to the wrist and hand flexors and extensors, substantiating our finding that wrist injuries were the most common injury [15]. Esports players may even be at a higher risk than other computer and console occupations as they perform up to 500-600 actions per minute (APMs), as defined by the number of keyboard, mouse, or console inputs, compared to 130-180 APMs that office workers perform [3,16,17]. Combining the increased APMs with more than five hours per day playing esports leads to excessive stress loading on distal wrist/hand tendons.

Apart from the lack of research on the prevalence of injuries, there has been a recent increase in studies on esports-related physiological and psychological effects. These studies have identified other injury risk factors in esports athletes such as the sedentary and non-active nature of esports, potentially higher body mass index, and decreased bone mineral content compared to non-esports players [18,19]. These risk factors for musculoskeletal injury help explain why injury rates are high despite the lack of trauma. Moreover, esports players have an increased risk of circadian rhythm changes and resultant insomnia from constant blue light emission, mood disorders such as anxiety and depression, and other mental health issues [20]. While these studies are important risk factors for musculoskeletal injuries, there must be a stronger research emphasis on the physical health of these athletes as the overall industry and number of college programs and scholarships are only trending upwards.

Limitations

There are several limitations to this study. First, it is a cross-sectional study without a control group, which limits the generalizability of the results and restricts the ability to draw conclusions on causality. Only 18 of the respondents were females, likely reflecting that females are a small minority in collegiate esports and potentially creating a male-dominated bias. Additionally, surveying only college esports athletes of mostly similar ages and experience levels could be a source of missed associations and significant results. The nature of a self-reported survey also lends itself to reporting bias as none of these injuries were reported in medical records or a regulated injury surveillance system. Similarly, there was a low response rate due to a lack of publicly available email addresses and phone numbers of student-athletes. While emails were sent out to most collegiate programs through a convenience sampling method, there is no assurance of complete randomization and elimination of bias. Finally, the survey did not include the hours per day question as a continuous variable, thus disallowing the calculation of a correlation coefficient between training hours per day and injury history.

## Conclusions

Collegiate esports players who trained for more hours per day (31.7% of injured players vs. 10.7% of uninjured players practicing for more than five hours/day, p = 0.01) and played competitive collegiate esports for more years (2.0 ± 1.0 vs. 1.7 ± 0.9 years, p = 0.03) were more likely to have experienced an esports-related injury. The three most common injury locations were to the wrist, back, and neck, while shoulder and thumb injuries were the least common. Fortunately, only a small portion of athletes who experienced an injury was forced to miss competition time, and even fewer required surgery. With this being the largest investigation into collegiate esports-related injuries, future medical research regarding the incidence, management, and prevention of its injuries can help collegiate and professional programs place a greater emphasis on the health of their athletes.

## Appendices

Table 4 represents the survey sent to the collegiate esports athletes and the answer choice options for each respective question. 

**Table 4:** Survey questions and answer choices that were sent to esports athletes. Empty cells represent questions that required a short answer response.

Survey questions	Answer choices
What is your current age?	
At what age did you begin participating in esports?	
Please select your sex	Male, Female, Prefer not to say
How long have you been a member of your school’s esports varsity team?	1, 2, 3, 4, 5 (years)
Are you currently on scholarship for your esports team?	Yes, No
Approximately how many days per week do you play esports?	1, 2, 3, 4, 5, 6, 7 (days)
Approximately how many hours per day do you play esports?	0–1, 1–2, 2–3, 3–4, 4–5, 5+ hours
Have you sustained any of the following injuries due to esports? Select all that apply	Thumb, Finger, Hand, Wrist, Elbow, Neck, Back, Shoulder, None
Have you required surgery for any of the above injuries?	Yes, No
Have you had to miss any esports competitions for any of the previous injuries?	Yes, No
Has an esports injury caused you to miss any time from playing esports?	Yes, No
If so, how much time (in weeks) did you miss?	
How significantly did the injury/injuries affect your esports playing?	Scale of 1–10 (1-not at all; 10-significantly worse quality of play)
Did you require surgery for any of the above injuries?	
